# Drivers, adaptations, and public impacts of hospital closures: implications for policy

**DOI:** 10.3389/fpubh.2024.1415033

**Published:** 2024-08-13

**Authors:** Soroush Saghafian

**Affiliations:** Harvard Kennedy School, Harvard University, Cambridge, MA, United States

**Keywords:** hospital closures, public health, public policy, health services, hospital management

## Abstract

A concerning number of hospitals have closed in the US in recent years and there are many other hospitals that are at significant risk of closure in the coming years. The COVID-19 pandemic magnified the trend of hospital closures, raising further concerns about the potential impacts of hospital closures and the important need for devising policies that can mitigate them. To devise such policies, however, we first need to better understand the main drivers, potential adaptations by providers, and the widespread public impacts of hospital closures. We also need to recognize various changes in care delivery modes and related practices. Understanding these complex issues can allow policymakers to shift their focus from the narrow scope of “access to care,” and instead take into account various other consequences of hospital closures that are currently largely overlooked but need to be part of policy discussions.

## 1 Introduction: main drivers of hospital closures

There are various drivers that contribute to the increasing trend of hospital closures in the US. The first driver is related to the financial challenges. Specifically, hospitals operate on tight margins and face financial pressure from a variety of sources, including reduced reimbursement rates from government payers like Medicare and Medicaid, uncompensated care for uninsured patients, and rising costs for supplies, technology, and labor. Financial challenges are particularly acute for rural hospitals, which typically have smaller patient populations and struggle to attract and retain physicians. Financial challenges were exacerbated for many hospitals during the COVID-19 pandemic, in part because they had to stop more profitable services (e.g., elective surgeries) and shift their resources toward less profitable COVID-19 patients.

The second driver is related to changes in healthcare policy. Healthcare policy changes—at the state or federal level—can have a significant impact on hospitals. For example, changes to Medicare reimbursement rates or eligibility criteria can affect hospital revenues. In parallel, states' Medicaid expansion decisions can affect the number of insured patients their hospitals serve, and hence, are known to be directly related to hospital closures (1, 2). The US government, however, has had a few mechanisms in place to provide funding for hospitals facing challenging circumstances. Examples include the Disproportionate Share Hospital (DSH) payment that supplements the cost of uncompensated care, the Critical Access Hospital (CAH) program aimed at covering the high fixed costs that rural hospitals often face, and the Consolidated Appropriations Act that introduced a new hospital type termed “rural emergency hospital” (REH).

These funding mechanisms, however, have been subject to various alterations (3, 4). For example, recent healthcare reforms have attempted to cut the DSH fund by $35.1B between FY2017 and FY2024, and there have been various efforts to make the CAH program's eligibility stricter (3). The COVID-19 pandemic, however, forced the US government to increase its support for hospitals that have been struggling. Specifically, the government provided additional federal support such as the Coronavirus Aid, Relief, and Economic Security (CARES) Act, devoting $175B for providers hit hard by COVID-19 (3). The Department of Health and Human Services has also expanded the Medicare Accelerated and Advance Payment program—a loan program that helps hospitals with disruptions in cash flow (3). These supporting mechanisms have enabled some struggling hospitals to “take a breath”, enabling them to acquire essential equipment, recoup lost revenues, and most notably, stay open. Nevertheless, funds are restricted and largely constrained. What is more, the US government in some cases—especially with regards to stimulus packages such as CARES Act—has not been able to implement effective mechanisms that ensure the funds go to hospitals that are at utmost need—and not the wealthier ones that did not face significant financial or operational challenges during the pandemic. Similarly, the recent Consolidated Appropriations Act that made over 1,500 rural hospitals eligible for funds through a REH designation—including more than $3.2 million in 2023 as additional facility payments—could help rural hospitals at risk of closure (5, 6), but has introduced various unintended consequences such as worsen access, increased rejected transfers and delays in care due to capacity limits, higher levels of care fragmentation, and reduced workforce across inpatient and outpatient settings (5).

The third driver of hospital closures is a rapid growth in industry consolidation and vertical integration. By and large, the healthcare industry has been consolidating at a rapid speed, with more powerful hospital systems acquiring smaller, independent hospitals. Similarly, medicine in the US is becoming more vertically integrated with larger hospitals acquiring independent physician practices (7). These concerning changes can lead to closures of smaller facilities that are no longer financially viable or competitive, or those that are deemed redundant by the larger healthcare systems.

The fourth driver is related to the demographic shifts. Changes in population demographics can also affect hospital viability in significant ways. For example, as populations age, there is an increased demand for certain types of healthcare services (e.g., long-term care), while other services may face a decreased demand. These changes in demand, caused by changes in population demographics, create significant issues for some hospitals, pushing them to close entirely or at least shut down some of their services. Some of the lock-down policy interventions implemented by the US government during the early stages of the COVID-19 pandemic [see, e.g., (8)] also reduced the mobility of some patients, making them less likely to travel to hospitals, changing the demographic portfolio of patients they serve.

Demographic shift among providers is also another contributing factor to some hospital closures. Specifically, in smaller and rural hospitals, there is a considerable trend of the physician workforce aging. This, coupled with high rates of provider turnover, poses significant challenges for such hospitals. For example, when a key hospital specialist (e.g., an obstetrician, general surgeon, or orthopedic surgeon) is lost due to retirement or difficulties in retaining them, the impact on the hospital's financial health can be severe, exacerbating its vulnerability to closure.

The fifth driver is the tendency of some patients, especially in rural areas, to not seek care at their closest hospital, a phenomenon known as “hospital bypass.” Some recent analysis indicates that about 50% of discharges from hospitals related to patients that bypass their closest hospital (9). This bypass behavior of patients, perhaps in hope for receiving higher quality of care, leads to reduced demand in local rural hospitals, imposing significant financial distress and spiked risk of closure (10, 11).

Finally, there are various technological advances that affect care delivery models and hospital operations. For example, the rise of telemedicine post COVID-19 has made it easier for patients to receive care remotely, reducing the need for in-person visits and potentially leading to lower demand for certain types of hospital services. Mobile Health (mHealth) technologies that are now more common than before—thanks to advancements in smart phones, sensors, and Internet of Things (IoT) enabled devices—have also reduced the need for patients to go to hospitals, thus, reducing the demand for some of their services (12). And a reduction in demand often has important revenue related consequences for hospitals, pushing some closer to closure. Beyond the impact on the size of the demand, however, technological advances along with various payment reforms, have created changes in the type of demand for hospital services. In particular, hospitals have faced significant shifts in demand from inpatient to outpatient care, impacting their revenues and risk of closure.

While the focus of this piece is the US, it is useful to also note that the equivalent of some of these drivers exist in other countries. Nonetheless, the US healthcare system is significantly different from that of many other countries, and thus, comparisons might not be meaningful. For example, the US healthcare system is strongly influenced by technological advancements in treatments, pharmaceuticals, and medical devices that often come with high R&D and implementation costs. In the US, these costs are typically passed on to patients, insurers, hospitals, and the healthcare system more broadly. In the single-payer healthcare systems, however, prices are more strongly negotiated, resulting in less direct increases in the costs. In the mixed public-private systems, a diverse set of government regulations, negotiations, and market mechanisms are employed, which also alter some of the driving forces discussed above.

Providing correct mechanisms for financial support to struggling hospitals, policy changes, incentivizing suitable integrations and mergers in support of less stable hospitals, and expanding telehealth services to areas affected by hospital closures (or making use of community-based healthcare solutions in such areas) are among the strategies that can mitigate the impacts of some the drivers of hospital closures. However, in addition to understanding these drivers, to better comprehend the impact of hospital closures, and thereby design more effective policies, we also need to consider how providers—remaining hospitals and physicians—adapt when a hospital closes. And this is where findings from various recent studies can particularly help.

## 2 Nearby hospitals adaptation to hospital closures

When a hospital closes its nearby hospitals face the challenge of handling a sizable demand spike, since the needs of patients of the closed hospital are added to their typical workloads. So how do such hospitals adapt to the spike demand?

A recent large-sale analysis of over 14 million patient visits across the US (4) suggested that the nearby hospitals on average improve their operational efficiency. That is, without adding much additional capacity, they end up serving more patients. However, they do so via a *speed-up* response. That is, by reducing their average service duration instead of lowering their average bed idle time. What is more, this speed-up response negatively affects some aspects of quality of care such as the 30-day mortality rate (4).

These “spillover” effects of hospital closures are largely overlooked, but are highly important to take into account by policymakers and other authorities who want to mitigate the negative consequences of hospital closures. This is especially important, considering that evidence suggests that the nearby hospitals cut some value-added care delivery steps in response to facing the spiked demand caused by a closure (4).

Moreover, it is equally important to note that these spillover effects of hospital closures are highly heterogeneous. For example, nearby hospitals that are often considered more desirable (e.g., high-quality, urban, and teaching hospitals) tend to experience more prominent spillover effects on their operations. Notably, these heterogenous effects often magnify social disparities by enlarging the existing efficiency gaps between the more and less desirable hospitals (3, 4).

Finally, nearby hospitals might alter some of their protocols and deviate from preferred patient-provider assignments in response to the increased demand. Hospitals operations in the US as well as patient outcomes and expenditures are already affected by such deviating behaviors (13), and hospital closures might intensify them.

## 3 Physicians adaptation to hospital closures

When a hospital closes, physicians who practice there may face a variety of challenges as they seek to adapt to the new circumstances. Besides understanding how hospitals respond to closures, it is vital to comprehend the response from physicians.

Overall, adapting to a hospital closure can be a complex and challenging process for many physicians, requiring a significant amount of patience, flexibility, and resilience, among other traits or behaviors of the physicians. Some of the most common ways for physicians to adapt—besides relocating to a new area or commuting to a different hospital—include exploring alternative practice models, considering opening a new practice, and retiring or leaving the profession.

Specifically, many physicians may explore alternative practice models, such as telemedicine or concierge medicine, that allow them to provide care outside of the traditional hospital setting. New internet-enabled technologies also allow them to be hired in location-independent roles, including serving as a tele-triage physician (14).

To adapt, some physicians may consider opening their own practice or joining a small group practice. This can provide greater autonomy and control over their work, but also comes with the burden of managing a business. In addition, for some physicians, a hospital closure may be a signal to retire or leave the profession altogether. This can be particularly true for physicians who are close to retirement, or those who are reluctant to start over in a new healthcare system. These changes can significantly alter the supply side of healthcare.

But can physicians easily keep their career going after their hospital closes? In some cases, there may be many opportunities available, and physicians may be able to transition to a new hospital or practice without much hassle. In other cases, it may be more exigent to find new employment opportunities, particularly in rural areas where there may not be any other hospital nearby. The ease of switching also depends on the current setting in which a physician is practicing. For example, some physicians already use telemedicine, tele-triage, or other related technological advancements to serve multiple hospitals, and hence, they may not face much trouble when one of their hospitals closes.

In addition, physicians' professional network and financial resources can also play a significant role in how they can respond to their hospital being closed. Thus, there are various individual level differences that can play a role as well.

It is also essential to note that hospital closures can have ripple effects throughout the healthcare system, and the impacts may not be limited to specific specialties. For example, primary care physicians who refer patients to the hospital for specialty care may have to refer them to different hospitals after a closure, which can impact their patient relationships and continuity of care.

Nonetheless, hospital closures often affect those specialties that rely heavily on hospital-based services more than others. For example, some hospitalists often work exclusively in the hospital setting and do not have any private practice outside of the hospital. When a hospital closes, hospitalists may have to find new jobs or relocate to another hospital. Emergency medicine physicians also often work primarily in a hospital emergency department (ED), although even in that case there are various inter-physician differences among them, including significant difference in key performance measures such as adjusted average length of stay (15), resource utilization, and admission rate (16, 17). When a hospital closes, its ED also closes, forcing its ED physicians to relocate to another hospital.

Another group of physicians that are often affected by closures are anesthesiologists who provide anesthesia services for patients undergoing surgery or other procedures in the hospital. When a hospital closes, there may be fewer opportunities for anesthesiologists to practice in the area due to the nature of their specialty. Similarly, hospital closures often affect OB/GYNs providers in charge of women during pregnancy, childbirth, and postpartum. When a hospital with a labor and delivery unit closes, OB/GYNs may also need to relocate. And the relocation of physicians can change demand vs. supply ratios, mainly because the patients move their healthcare needs “locally”, while physicians might move out of state or relocate to further locations. Such changes in demand vs. supply can, in turn, have various large-scale negative impacts.

## 4 Conclusion

Despite various available studies (see, e.g., (18) for a review), more research is needed to get a better picture of the entire impact of hospital closures. But what we know is that their impact goes well-beyond the typical discussion on patients' access to care. Notably, hospital closures have serious impacts on providers—physicians and the nearby hospitals. Limiting the discussion to access to care is not only a disservice to providers and the entire healthcare sector, but could also mislead potential policy solutions.

For example, understanding that a hospital closure can induce speed-up behavior in a nearby hospital, and that this speed-up behavior in turn can negatively affect the quality of care delivered there, offers a new policy perspective. Should policymakers direct resources to bail out hospitals that if closed would impact access to care, or should they instead bail out hospitals that if closed would significantly impact the quality of care of their nearby hospitals? Similarly, understanding the adaptation by physicians can encourage policymakers to direct resources to bail out hospitals that if closed would create large regional demand vs. supply mismatches. And there are various policy levers beyond bailout as well. For example, realizing that a hospital closure can induce speed-up behavior among nearby hospitals suggests yet another effective policy lever: monitoring length of stay changes post-closure among nearby hospitals. Overall, these aspects of hospital closures have been largely overlooked and more research is needed to provide policymakers with actionable insights.

Finally, shedding light on the less understood but widespread impacts of hospital closures can lead to better data collection efforts—a vital requirement for more intelligent policy designs. Given the increasing attention to campaigns aimed at collecting and publicly reporting hospital data, such as those in public and private public reporting efforts (19, 20), researchers and policymakers can focus on collecting more relevant data useful for predicting closures. This will enable them to make use of relatively accurate estimates of closure risks and obtain more actionable insights.

## Data Availability

The original contributions presented in the study are included in the article/supplementary material, further inquiries can be directed to the corresponding author.
